# Discovery of High-Risk Clinical Factors That Accelerate Brain Aging in Adults: A Population-Based Machine Learning Study

**DOI:** 10.34133/research.0500

**Published:** 2024-10-21

**Authors:** Jing Sun, Luyao Wang, Yiwen Gao, Ying Hui, Shuohua Chen, Shouling Wu, Zhenchang Wang, Jiehui Jiang, Han Lv

**Affiliations:** ^1^ Department of Radiology, Beijing Friendship Hospital, Capital Medical University, Beijing, China.; ^2^ Institute of Biomedical Engineering, School of Life Sciences, Shanghai University, Shanghai, China.; ^3^ Department of Radiology, Kailuan General Hospital, Hebei, Tangshan, China.; ^4^ Department of Cardiology, Kailuan General Hospital, Hebei, Tangshan, China.

## Abstract

**Introduction:** Brain age prediction using neuroimaging data and machine learning algorithms holds significant promise for gaining insights into the development of neurodegenerative diseases. The estimation of brain age may be influenced not only by the imaging modality but also by multidomain clinical factors. However, the degree to which various clinical factors in individuals are associated with brain structure, as well as the comprehensive relationship between these factors and brain aging, is not yet clear. **Methods:** In this study, multimodal brain magnetic resonance imaging data and longitudinal clinical information were collected from 964 participants in a population-based cohort with 16 years of follow-up in northern China. We developed a machine learning-based algorithm to predict multimodal brain age and compared the estimated brain age gap (BAG) differences among the 5 groups characterized by varying exposures to these high-risk clinical factors. We then estimated modality-specific brain age in the hypertension group based on hypertension-related regional imaging metrics. **Results:** The results revealed a significantly larger BAG estimated from multimodal neuroimaging in subjects with 4 or 5 risk factors compared to other groups, suggesting an acceleration of brain aging under cumulative exposure to multiple risk factors. The estimated T1-based BAG exhibited a significantly higher level in the hypertensive subjects compared to the normotensive individuals. **Conclusion:** Our study provides valuable insights into a range of health factors across lifestyle, metabolism, and social context that are reflective of brain aging and also contributes to the advancement of interventions and public health initiatives targeted at the general population aimed at promoting brain health.

## Introduction

While chronologically aging, the human brain undergoes a series of structural changes, including the atrophy of brain tissue volume [1], impairment of white matter (WM) microstructure [2], and a higher burden of white matter hyperintensity (WMH). These aging-related changes in brain macrostructure and microstructure have been considered to be associated with cognitive decline [3–5] and may increase the risk of neurodegenerative diseases such as dementia [4].

While the senescent neurodegenerative changes in the brain are widely recognized, the elderly populations often exhibit significant heterogeneity in the process of neurobiological aging [6]. Brain age, which represents the biological age of an individual’s brain [7], has been recognized as a reliable biomarker for assessing differences in brain aging among individuals. Machine learning algorithms have demonstrated great potential in accurately estimating an individual’s brain age by utilizing structural and functional characteristics obtained from brain magnetic resonance imaging (MRI) [8]. In addition, a variety of machine learning algorithms have been validated for precise estimation of brain age, such as relevance vector regression and Gaussian process regression [9]. The disparity between the estimated brain age and the chronological age of an individual, known as the brain age gap (BAG) [10], particularly when it exceeds zero, serves as a potential predictor of accelerated brain deterioration that may be associated with neurodegenerative diseases [11,12].

Currently developed brain age prediction models mostly utilize a single neuroimaging modality, with the most commonly used being structural features derived from T1-weighted imaging (T1WI) computed at the global or regional level [13]. In addition, whole-brain and tract-specific WM metrics derived from diffusion tensor imaging (DTI) also exhibit significant value in predicting brain age [14]. With the increasing availability of neuroimaging data, there is a growing imperative to integrate multiple modalities for comprehensive assessment of both structural and functional changes in the process of brain aging. The combination of information from multiple modalities provides a more comprehensive elucidation of individual differences in brain aging mechanisms and also enhances the accuracy of predicting brain age [15].

Furthermore, not only the imaging modality but also multidomain health factors play an important role in the degeneration of the brain. Accumulating evidence has suggested the potential association between multidomain health factors and neuroimaging metrics. For instance, exposure to alcohol consumption [16] and cigarette smoking [17,18], as well as higher body mass index [19–21], blood pressure [22], and diabetes status [23], are found to be negatively associated with brain structures. However, the extent to which these health factors are related to brain structures in individuals and the integrated relationship of these health factors with brain aging remains unknown. Therefore, it is essential to identify the informative health factors for the tasks of brain age prediction.

**Fig. 1.**
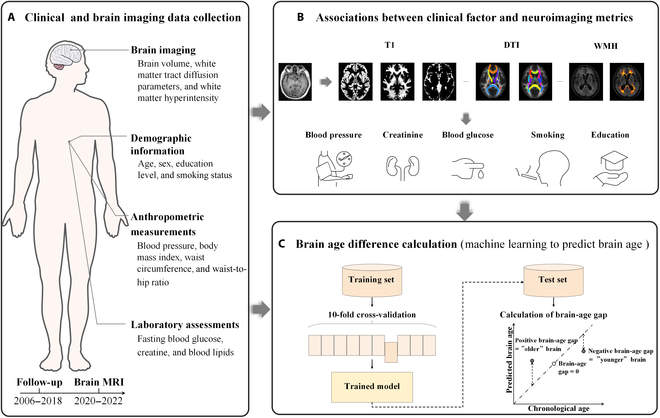
The schematic overview of the research design. (A) The data used in the study include brain imaging, demographic information, anthropometric measurements, and laboratory assessments. (B) Associations between clinical factors and neuroimaging metrics. (C) Brain age difference calculation based on machine learning.

In this study, we aimed to investigate how the multidomain health factors contribute to the degeneration of the brain structures in adult population. To this end, we firstly extracted the most informative clinical factors that exhibit the strongest associations with multimodal neuroimaging features. Then, we investigated whether machine learning algorithms integrating multimodal neuroimaging information could predict brain aging differences among different risk-exposure groups stratified by the amount of high-risk clinical factors. More specifically, given the intricate associations of not only the absolute blood pressure level but also the cumulative level and variability of blood pressure with brain structural changes [22,24,25], we hypothesized that blood pressure ranks undoubtedly one of the most informative clinical factors. Next, we respectively estimated modality-specific T1WI-derived and DTI-derived brain ages in the hypertension group based on hypertension-related regional imaging metrics. Based on previous findings, we expected to observe a larger estimated brain age in the high-risk-exposure group, as well as in the hypertension group. Figure 1 shows the schematic summary of the study design.

## Results

Prior to the analysis, subjects with incomplete measured data in critical imaging modalities—T1, DTI, and fluid-attenuated inversion recovery (FLAIR) (for WMH calculation)—were excluded. The final analysis concentrated on subjects with complete data for the 5 key risk factors identified through our correlation analysis. Cases lacking data on these critical factors were also excluded. Therefore, a total of 964 participants were included in the analysis, with 473 (44.0%) being female. The chronological age of participants in our study was calculated at the time of brain MRI examination. The median (interquartile range) age of participants was 55.0 (46.0, 64.75) years. We collected 254 clinical variables consisting of demographic information, anthropometric measurements, and laboratory assessments. A total of 1,272 neuroimaging metrics, including 1,122 DTI features, 120 T1WI features, and 30 WMH features, were extracted from the study population.

### Selection of most informative clinical factors

Through Pearson correlation analysis with false discovery rate correction, we identified the top 25 clinical variables that were regarded to be most closely associated with neuroimaging features (Table S1). These 25 risk factors included age at MRI acquisition, age of hypertension diagnosis, Montreal Cognitive Assessment (MoCA) delayed recall score, total MoCA score, smoking, education, sex, systolic blood pressure (SBP) 2014, SBP 2010, cumulative SBP during 2006 to 2018, chronological year of hypertension diagnosis, years of hypertension history, SBP 2012, cumulative SBP during 2010 to 2018, mild cognitive impairment (MCI) or non-MCI, fasting blood glucose (FBG) 2008, cumulative creatinine (Cr) during 2006 to 2018, cumulative Cr during 2010 to 2018, FBG 2006, MoCA abstraction score, cumulative Cr during 2014 to 2018, FBG 2010, Cr 2010, MoCA language score, and Cr 2014, according to the order of stronger to weaker correlation with neuroimaging.

**Fig. 2.**
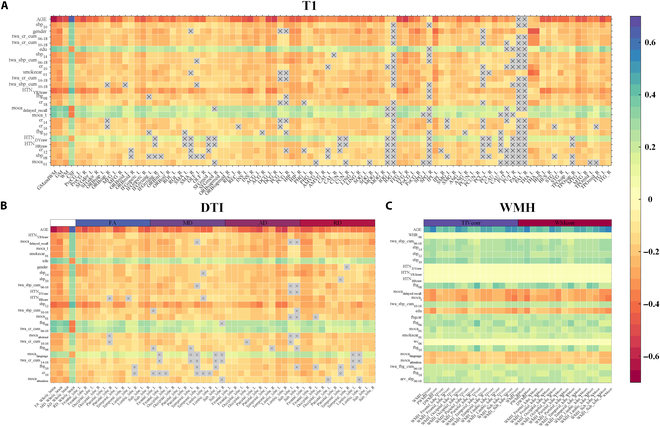
Pearson correlation of clinical variables with brain imaging metrics. (A to C) The top 10% clinical parameters most closely associated with neuroimaging features of T1, DTI, and WMH. GM, gray matter; FA, fractional anisotropy; MD, mean diffusivity; AD, axial diffusivity; RD, radial diffusivity.

We conducted a summary analysis of the factors with a relatively high proportion in the top 25 clinical variables. The aforementioned variables were mainly classified into 5 high-risk clinical factors, including blood pressure, Cr, FBG, smoking status, and education level. Furthermore, we also performed the correlation analysis between all clinical variables and T1WI metrics, DTI metrics, and WMH metrics separately. The results aligned closely with the primary analysis (Tables S2 to S4 and Fig. 2), indicating that the 5 high-risk factors were most strongly associated with neuroimaging markers.

Given the well-established significant correlation between age, sex, and brain aging, the 2 demographic factors were not prioritized in our research focus. Furthermore, in the brain age prediction model, we have controlled for the covariates of chronological age and sex.

### Multimodal brain age prediction

**Fig. 3.**
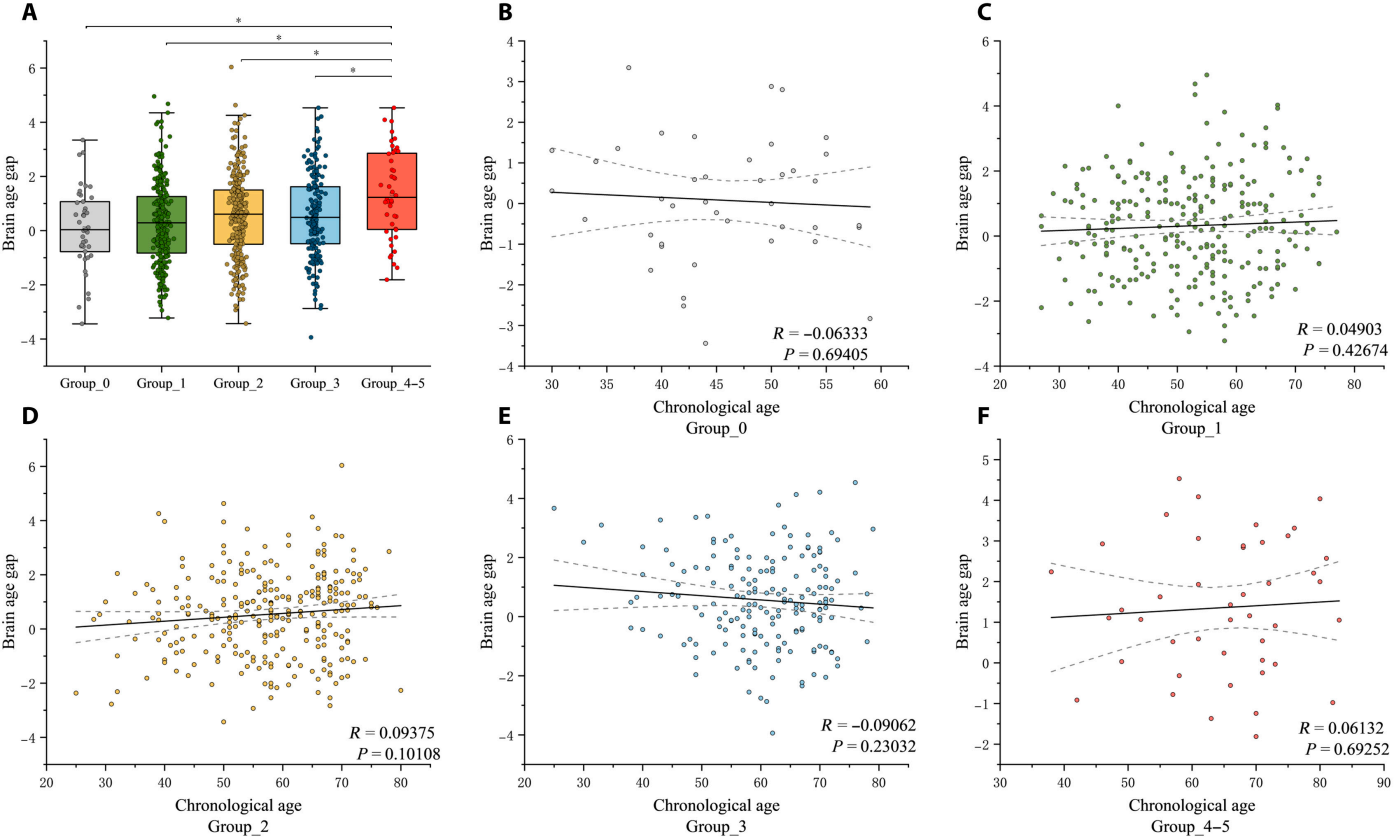
Regression plots of corrected BAGs and chronological age in different risk-exposure groups. (A) Corrected BAGs in normal group and different risk-exposure groups. The corrected BAGs in 1, 2, 3, and 4 to 5 high-risk factor groups were compared with those of normal individuals with 0 high-risk factor using one-way ANOVA and the Holm–Šídák multiple comparisons test. * indicates significance (*P* < 0.05). (B to F) Regression plots of corrected BAGs and chronological age in groups with 0, 1, 2, 3, and 4 to 5 high-risk factors, respectively. In each panel, the black solid line represents a regression line, and the dotted lines represent the 95% confidence intervals.

The performance of the machine learning models was evaluated using a comprehensive set of metrics, including mean absolute error (MAE), root mean square error, the square of Pearson’s correlation coefficient (*R* value) between chronological age and predicted brain age, as well as the mean delta age. In the normal test samples, the MAEs for Least Absolute Shrinkage and Selection Operator, Ensemble Trees, Deep Neural Network, and K-Nearest Neighbor models were 2.686, 1.099, 3.789, and 3.298 years, respectively. The *R*-squared (*R*^2^) values between chronological age and predicted brain age were 0.839, 0.960, 0.751, and 0.854, respectively (Table S5). Thus, the highest prediction accuracy was achieved by the Ensemble Trees model (MAE = 1.099 years, root mean square error = 1.875 years, *R*^2^ = 0.960, mean delta age = 0.214 years). Based on these results, we applied the Ensemble Trees model to different risk-exposure groups for brain age prediction. Figure 3 illustrates scatter plots of the correlation between estimated BAG (uncorrected and corrected) and chronological age in the 4 groups divided by the presented number of high-risk factors. We corrected brain age using coefficients identical to those for normal group. Intriguingly, the BAGs estimated from multimodal neuroimaging features in subjects with 4 to 5 high-risk factors were significantly higher compared to other groups with 0, 1, 2, and 3 high-risk factors (group 4 to 5 = 1.358, group 0 = 0.082, group 1 = 0.311, group 2 = 0.538, group 3 = 0.559, *P* < 0.05, Holm–Šídák test). However, the estimated BAG did not exhibit a difference across the groups with 0, 1, 2, and 3 high-risk factors.

### Modality-specific brain age prediction

**Fig. 4.**
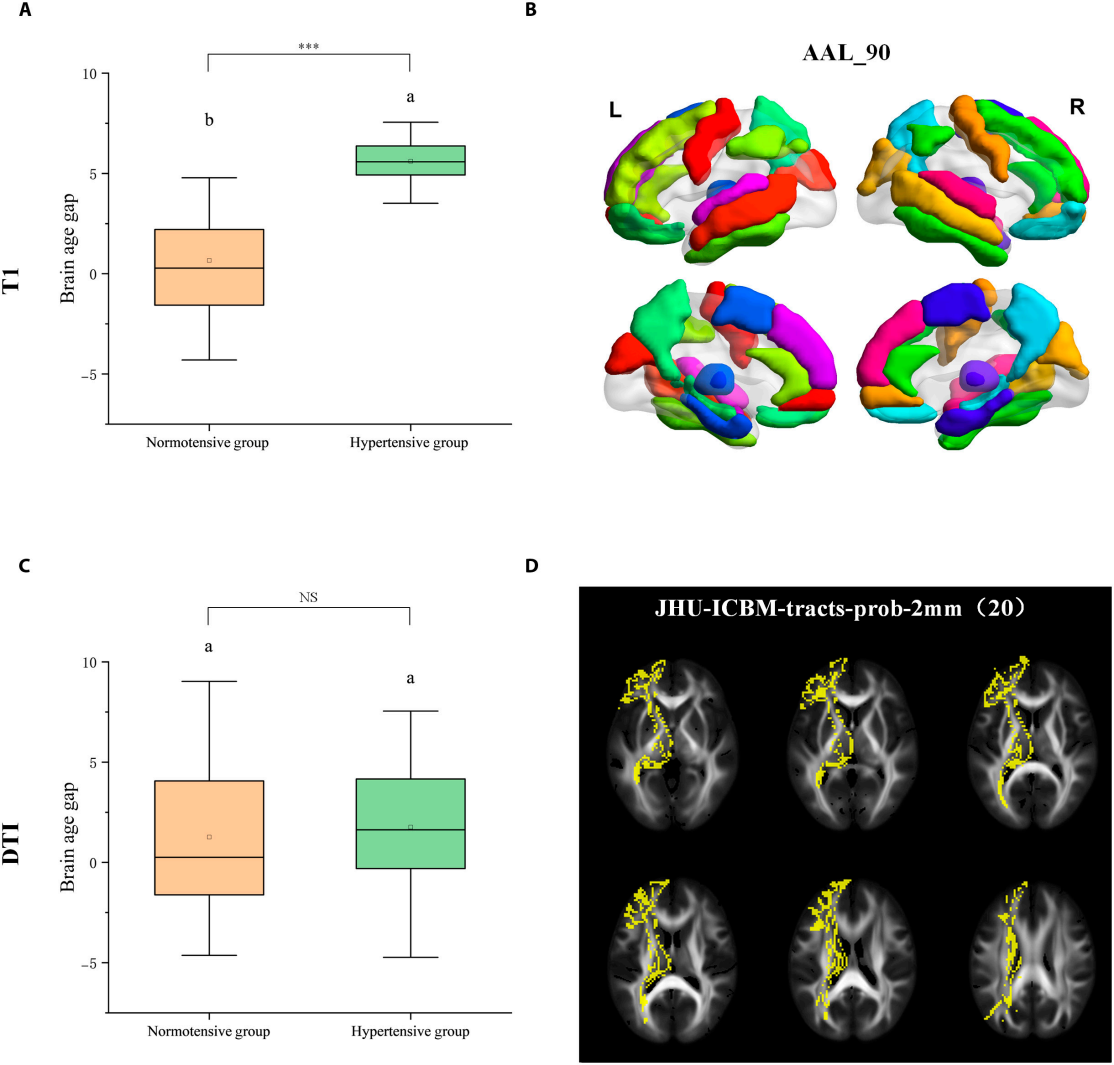
Corrected BAGs in normotensive and hypertensive groups. (A and C) Histogram of T1-based and DTI-based BAGs in normotensive and hypertensive groups. *** indicates significance (*P* < 0.0001), and NS indicates nonsignificance (*P* > 0.05). The *P* values were tested by Fisher’s least significant difference test. (B) The hypertension-sensitive sub-brain regions based on the AAL_90 atlas. (D) The hypertension-sensitive WM tracts based on the Johns Hopkins University (JHU)-International Consortium of Brain Mapping (ICBM)-tracts-prob 2mm.

According to the results of the correlational analysis, it was found that blood pressure exhibited the strongest association with neuroimaging features. The sensitive T1 and DTI subregional metrics were derived from the dataset. The modality-specific models for brain age prediction were established based on T1 and DTI metrics separately (Fig. 4). The estimated T1-based BAG was significantly higher in the hypertensive subjects compared to those normotensive subjects (BAG: 0.661 versus 5.609, *P* < 0.0001). However, there was no substantial difference in the estimated BAG using DTI metrics between hypertension and normotension groups (BAG: 1.267 versus 1.760, *P* = 0.508).

## Discussion

This study attempted to predict the increased risk of brain aging in populations without a diagnosis of life-threatening conditions through clinical risk stratification. In order to identify individuals at high risk of brain aging, our study first identified 5 most informative clinical risk indicators, including blood pressure, FBG, Cr, smoking, and education. Subsequently, we utilized several machine learning models to predict multimodal brain age and compared the estimated BAGs among the 5 groups characterized by varying exposures to these risk indicators. The results from the prediction model revealed a significantly larger BAG estimated from multimodal neuroimaging in subjects with 4 or 5 risk factors compared to other groups, suggesting an acceleration of brain aging under cumulative exposure to multiple risk factors. The primary findings suggest that a range of health factors across lifestyle, metabolism, and social context may collectively contribute to the accelerated degeneration of the brain.

Given that the established model was based on the integration of T1WI, DTI, and FLAIR metrics, we assessed the impact of various high-risk clinical factors on individual brain structures across the whole brain, and systemic accelerated brain aging was observed under the exposure to cumulative multiple risk factors. However, the impact of individual risk factors on the refinement of the brain age prediction model varies across diverse modalities and neuroanatomical structures [26,27]. In modality-specific analysis, subjects with hypertension were characterized as having higher estimated modality-specific brain age than normotensive subjects. This finding indicates that hypertension exerts a pivotal influence on the structural degradation of brain tissues, thereby accelerating the pathological degeneration of brain structures. In line with a previous study indicating older-appearing brains in individuals with higher blood pressure [28], our study demonstrates a credible and interactive association between higher cardiometabolic risk and accelerated brain aging. However, our study did not detect a larger BAG based on WM microstructural measures. Further investigation into the neurobiological mechanisms through which elevated blood pressure influences brain aging is expected to evaluate the differential effects of hypertension on the gray matter and WM.

This study has several strengths. Firstly, the participants were recruited from a large community-based multicenter cohort study that was prospectively conducted in the central region of the Bohai Sea Gulf. The findings are highly generalizable to the broader population in Northern China. Secondly, the participants have been followed up for 12 years prior to brain MRI examination. Through repeated measurements of a variety of kinds of clinical data including physical examinations, laboratory assessments, and life habits for multiple times, this research achieved objective and precise clinical data expansion, making the results of the correlation exploration more robust and the screening of high-risk clinical factors more accurate. In addition, it also effectively reduced possible random errors present in studies employing a cross-sectional design and single-time point measurement. Thirdly, as the included participants in this study cover a wide age range of adulthood, we conducted the correction of brain age estimation to mitigate potential bias arising from the nonlinear association between the estimated BAG and chronological age. Fourthly, we separately employed predictive models using multimodal and single-modal neuroimaging information to assess the impact of multidomain high-risk clinical factors and specific single high-risk factors on the structural brain age. Lastly, based on our research findings, it is promising that real-time monitoring of high-risk factors for brain aging based on wearable devices may be achieved in the future. Furthermore, the individuals could be alerted in time if abnormalities in these high-risk factors are identified during physical examinations.

However, there are also areas that need improvement. Firstly, although the study utilizes follow-up clinical data, all the subjects underwent only a single brain MRI examination. The absence of longitudinal brain MRI data limits the ability to assess dynamic changes in neuroimaging markers. Future studies should consider incorporating longitudinal brain MRI data to provide a more detailed understanding of brain aging processes. Secondly, while our study effectively uses T1 and DTI data, future research should aim to include more imaging modalities, such as functional MRI, to provide a more comprehensive view of brain aging. Additionally, the extraction of quantitative measures from the original neuroimaging data might reduce the richness of the information. Future research should aim to utilize high-dimensional multimodal neuroimaging data to preserve more detailed information and improve predictive power [29]. Expanding the geographic and demographic diversity of the study sample could also improve the generalizability of the findings. Future studies should aim to include a more diverse population to ensure the results are applicable to a broader demographic.

In conclusion, our study indicates that the subjects under cumulative exposure to multiple risk factors exhibit a significantly larger BAG using multimodal neuroimaging information, suggesting an acceleration of brain aging. Additionally, hypertension is associated with a higher estimated modality-specific brain age compared to normotensive subjects. Our study may help promote healthy brain aging process in the general population by providing valuable insights into a range of risk factors across metabolism, lifestyle, and social background. Our findings not only enhance public awareness regarding the significance of maintaining brain health but also contribute to the advancement of interventions targeting these specific risk factors and public health initiatives aimed at enhancing brain health.

## Materials and Methods

### Participants

The present study was embedded in the Kailuan Study, a population-based prospective cohort study initiated in northern China since 2006 [30]. Demographic questionnaires, physical examinations, and laboratory assessments were conducted every 2 years at 11 local hospitals. In December 2020, the multimodality MEdical Imaging sTudy bAsed on KaiLuan Study (META-KLS) was launched as a subset of the Kailuan Study to recruit adult individuals for brain MRI examinations to evaluate brain health [31].

In this study, the inclusion criteria for participants were subjects who met all of the following: (a) acquired once brain MRI data during 2020 and 2022; (b) attended more than 3 clinical visits, during which pertinent clinical data were collected; and (c) without clinical diagnosed stroke, dementia, or neuropsychiatric disorders. The exclusion criteria were as follows: (a) missing data in brain imaging modalities; (b) missing information on age and sex; and (c) a history of cancer. The META-KLS has been approved by the Medical Ethics Committee of the Kailuan General Hospital. Written informed consent was obtained from each participant.

### Clinical feature collection and calculation

The demographic information (age, sex, education, smoking and alcohol consumption status, and physical exercise), anthropometric measurements (body mass index, waist and hip circumference, and systolic and diastolic blood pressure), and laboratory assessments (FBG, total cholesterol, high-density and low-density lipoprotein cholesterol, triglycerides, neutrophil count, Cr, and uric acid [UA]) of participants for 7 visits from 2006 to 2018 were collected from the Kailuan dataset. A binary feature of low risk or high risk was labeled to each clinical feature according to the above criteria. The definition of clinical characteristics at high risk was as follows: low education, high school and below; smoking, former or current smokers; alcohol consuming, former or current drinkers; self-reported physical exercise (rather than assessed with wearable device) [32], less than 3 times/week for ≥30 min; overweight or obesity, body mass index ≥ 24.0 kg/m^2^; abdominal adiposity, waist circumference ≥ 85 cm in men and ≥ 80 cm in women; abdominal obesity, waist-to-hip ratio ≥ 0.90 in men and ≥ 0.85 in women; hypertension, SBP ≥ 140 mmHg and (or) diastolic blood pressure ≥ 90 mmHg, taking antihypertensive medications, or self-reported hypertension history; diabetes, FBG ≥ 7.0 mmol/l, taking hypoglycemic medications, or self-reported diabetes history; hyperlipidemia for total cholesterol, ≥5.2 mmol/l; hyperlipidemia for high-density lipoprotein cholesterol, <1.0 mmol/l; hyperlipidemia for low-density lipoprotein cholesterol, ≥3.4 mmol/l; hyperlipidemia for triglycerides, ≥1.7 mmol/l; neutropenia or neutrophilia, neutrophil count < 1.8 or > 6.3 mmol/l; hypercreatininemia, Cr ≤ 97 μmol/l in men aged between 20 and 59 years, ≤ 111 μmol/l in men aged between 60 and 79 years, or ≤ 73 μmol/l in women aged between 20 and 59 years, ≤ 81 μmol/l in women aged between 60 and 79 years; hyperuricemia, male UA levels > 7 mg/dl (420 μmol/l) and female UA levels > 6 mg/dl (360 μmol/l). Otherwise, feature of low risk was labeled. Moreover, due to the unavailability of data on sleep duration [33] and humoral and cellular immune responses [34], these variables were not incorporated into the analysis.

In addition, the cumulative [25] and variability [24] characteristics of clinical variables are also key features that are strongly correlated with neuroimaging markers. The cumulative indicator reflects the cumulative value of a risk exposure over a long period of time, which is an important concept in health risk assessment. Additionally, variability reflects long-term fluctuations and changes in clinical parameters. In order to comprehensively depict the dynamic characteristics of variables, the cumulative and variability parameters of anthropometric measurements and laboratory assessments during 2006 to 2018, 2010 to 2018, and 2014 to 2018 were separately calculated. Specifically, the cumulative parameter was recorded as time-weighted average value, calculated by adding up the values of 2 consecutive examinations beginning with the first examination and dividing the total value by the years of follow-up. For variability assessment, 2 widely used statistical approaches were employed. The average real variability was expressed as the sum of the absolute differences in every 2 sequential measurements, divided by the number of measurements minus 1. The coefficient of variation was computed by dividing the standard deviation of all measurements by the mean value. Therefore, the cumulative and variability characteristics of clinical variables, in conjunction with cross-sectional characteristics, may facilitate better classification of different high-risk groups.

The cognitive function was evaluated using the MoCA on the same day of brain MRI acquisition. The scores in total and 7 cognitive domains (visuospatial/executive, naming, attention, language, abstraction, delayed recall, and orientation) were recorded. Subjects with less than 26 points were featured as MCI.

### Brain MRI acquisition and quantification

All brain MRI examinations were performed using a single 3.0-T MR scanner (GE Healthcare 750W, Milwaukee, Wisconsin, USA) with an 8-channel phased-array head coil at Kailuan General Hospital to ensure the reliability and comparability of the imaging data. Structural brain MRI included the high-resolution T1WI, DTI, and 3-dimensional FLAIR sequences. The parameters were listed as follows. T1WI: bandwidth = 41.67 KHz, flip angle = 15°, slice thickness/space = 1 mm/1 mm, repetition time = 6.7 ms, echo time = 2.6 ms, acquisition matrix = 256 × 256, field of view = 25.6 × 25.6 cm^2^, number of excitation = 1; DTI: bandwidth = 250 KHz, flip angle = 90°, slice thickness/space = 5 mm/5 mm, repetition time = 8,000 ms, echo time = 97.9 ms, acquisition matrix = 128 × 130, field of view = 24 × 24 cm^2^, number of excitation = 2; FLAIR: bandwidth = 62.5 KHz, flip angle = 90°, slice thickness/space = 1 mm/1 mm, repetition time = 5,000 ms, echo time = 1,147 ms, acquisition matrix = 256 × 256, field of view = 25.6 × 25.6 cm^2^, number of excitation = 1.

The brain macrostructural volumetric features were extracted on high-resolution T1WI using the Statistical Parametric Mapping 12 software and CAT12 package (http://www.neuro.uni-jena.de). We calculated the total intracranial volume (TIV) and the absolute volume of total gray matter, WM, and cerebrospinal fluid of each subject. The volumes of sub-brain regions were also calculated according to the AAL_90 (Automated Anatomical Labeling) atlas. These volumetric measurements were then normalized to individual head size and expressed as the percentages of TIV.

The brain microstructural analyzes based on DTI sequence were performed using a standardized and validated processing workflow [31]. Microstructural integrity measures at the global-brain level included fractional anisotropy, mean diffusivity, axial diffusivity, and radial diffusivity. In addition, the analysis of sub-brain level included diffusion metrics calculation with Johns Hopkins University (JHU) labels atlas and JHU tractography atlas, tract-based spatial statistics analysis, and WM tracts analysis with XTRACT pipeline.

The quantification of WMH volume was performed utilizing the Lesion Prediction Algorithm based on 3-dimensional FLAIR sequences, as well as the deep WMH and periventricular WMH. The WMH distribution along the WM tracts was also analyzed with the method of tract-based spatial statistics analysis. The results of WMH quantification were then corrected for TIV and WM volume.

### Correlation analysis of clinical features with neuroimaging metrics

The associations of various types of clinical variables with brain imaging metrics were investigated with Pearson correlation analysis via the MATLAB R2018b. We examined these associations based on the quantitative features extracted from multimodal brain MRI. The statistical significance thresholds were set at *P* < 0.05 after false discovery rate correction. The number of neuroimaging features indices significantly correlated with clinical factors was counted and ranked in descending order. The top 10% clinical parameters were considered to be most closely associated with neuroimaging features. These significant parameters were then classified into characteristics associated with a specific clinical risk factor (e.g., blood pressure).

### Machine learning-based brain age prediction

#### Multimodal brain age prediction in different risk-exposure groups

Participants who were free of high-risk clinical factors were defined as the normal group. The normal group was then randomly divided into training and testing sets at a ratio of 8:2. Through a 10-fold cross-validation conducted on the training set, the model exhibiting the smallest error in each fold in the validation set was selected for testing on the testing set, and the testing error was calculated. The performance of the final model was represented by the mean error of the selected model in each fold on the testing set. Finally, the MAE feature was computed to assess the accuracy of brain age prediction. The Spearman correlation analysis between predicted brain age and chronological age was also performed to test the consistency of brain age prediction.

To validate the robustness of the results, we compared the performance of various machine learning models in predicting brain age and conducted experimental investigations with 4 classical regression algorithms: Least Absolute Shrinkage and Selection Operator, Deep Neural Network, K-Nearest Neighbors, and Ensemble Trees. As the number of analyzed features in the study exceeded the number of samples, principal component analysis was employed for dimensionality reduction while preserving the most informative features in the dataset [35]. The machine learning model demonstrating the highest predictive accuracy was utilized for the task of brain age estimation.

We examined the individual differences in the interaction between chronological aging and high-risk clinical factors through the computation of BAG metrics. In addition, the estimated BAG generally shows a correlation with chronological age. However, due to regression dilution, younger individuals tend to have overestimated BAGs, while older individuals tend to have underestimated BAGs [36]. Therefore, we corrected the BAG for age bias using the linear bias correction method descried by Smith et al. [37].

Defining *x* as the actual age and *y* as the predicted age, the BAG was calculated as:

BAG=y−x (1)

The BAG was corrected for age bias by fitting a linear regression of *y* on *x*, represented as:

y=ax+b (2)

By fitting correction formula to the test data set, the corrected brain age gap *BAG_c_* can be calculated using:

$${\mathrm{BAG}}_{c}=\frac{y-b}{a}-x$$ (3)

The coefficients *a* and *b* derived from the normal group were used to correct for bias in the same way when applied to the different risk-exposure groups.

According to the number of labeled high risk for blood pressure, Cr, FBG, smoking status, and education level, all the participants were categorized into 5 groups based on the presence of having 0, 1, 2, 3, and 4 to 5 high-risk clinical factors (0 high-risk factor [normal], *n* = 203; 1 high-risk factor, *n* = 265; 2 high-risk factors, *n* = 277; 3 high-risk factors, *n* = 180; 4 to 5 high-risk factors, *n* = 39). Thus, these 5 groups were characterized by varying exposures to these high-risk clinical factors, stratified by the amount of high-risk clinical factors.

We constructed the brain age prediction model in 161 normal subjects using multimodal brain MRI features, including whole-brain and regional volumetric measures derived from T1WI, whole-brain and tract-specific WM metrics derived from DTI, and WMH volume derived from FLAIR. The generated brain age prediction model was applied to groups of presenting 1, 2, 3, and 4 to 5 high-risk clinical factors separately for BAG estimation.

#### Modality-specific brain age prediction in the hypertension group

In addition, based on the results of correlation analysis, we identified blood pressure as the clinical factor exhibiting the strongest correlation with neuroimaging features. Hence, we performed modality-specific T1-based and DTI-based brain age prediction analysis in the subjects with hypertension. Based on a review of the published evidence, we identified sensitive brain regions associated with hypertension. The analyzed features include the volume of sub-brain regions and the diffusion characteristics of water molecules in WM tracts. The hypertension-sensitive sub-brain regions based on the AAL_90 atlas refer to the thalamus, hippocampus, cuneus, amygdala, inferior parietal lobules, precuneus, parahippocampal gyrus, superior and middle temporal gyrus, superior and medial frontal gyrus, precentral gyrus, inferior temporal gyrus, supplementary motor areas, and anterior cingulate cortex [38–41]. The hypertension-sensitive WM tracts based on the JHU labels and tractography atlas include inferior fronto-occipital fasciculi, superior and inferior longitudinal fasciculus, uncinate fasciculus, splenium, anterior thalamic radiation, forceps minor, and forceps major [39,42–44]. The prediction models are constructed based on the extracted continuous values.

Excluding the confounding effects of other risk factors (i.e., labeling for high-risk serum Cr, FBG, smoking, and education was all zero), participants were categorized into 2 groups based on the presence of hypertension (hypertension, *n* = 203; no hypertension, *n* = 89). The brain age model, constructed using multimodal brain MRI features from 161 normal subjects, was applied to the hypertension group for BAG estimation.

### Statistical analysis

Statistical analyses were conducted using Origin 2024b (https://www.originlab.com/2024bC). The corrected BAG in participants grouped by the number of high-risk clinical factors was compared to that in the normal group using one-way analysis of variance (ANOVA) and the Holm–Šídák multiple comparisons test. In addition, the BAG was compared between the hypertension risk group and the no-risk group using Fisher’s least significant difference test. A *P* value <0.05 was considered statistically significant. 

## Data Availability

Clinical data will be available for other research groups whose proposed use of the data has been approved by an independent review committee identified for this purpose. Requests for data should be directed to the principal investigator, Dr. Z.W. (cjr.wzhch@vip.163.com).
